# Boys and girls differ in their rationale behind eating: a systematic review of intrinsic and extrinsic motivations in dietary habits across countries

**DOI:** 10.3389/fnut.2023.1256189

**Published:** 2023-09-29

**Authors:** Alysha L. Deslippe, Coralie Bergeron, Tamara R. Cohen

**Affiliations:** ^1^Faculty of Land and Food Systems, University of British Columbia, Vancouver, BC, Canada; ^2^Healthy Starts, British Columbia Children's Hospital Research Institute, Vancouver, BC, Canada; ^3^Faculty of Medicine, University of British Columbia, Vancouver, BC, Canada

**Keywords:** dietary behaviors, adolescence, gender, self-determination theory, motivation, health promotion

## Abstract

**Background:**

Boys' and girls' food habits diverge in adolescence (13–18 years). This contributes to unequal risks of adverse health outcomes based on sex and gender in adulthood (e.g., heart diseases in men vs. disorder eating in women). Though multi-factorial, why these dietary differences occur is unclear.

**Purpose:**

To identify the reasons why adolescents' motivation behind dietary habits differs among genders.

**Methods:**

Four databases were searched following PRISMA guidelines. Eligible studies had to use qualitative methodology and report at least one gender unique theme. Reported themes were thematically analyzed, with a sub-analysis by country where the studies were conducted. Quality appraisals were assessed using the Critical Appraisal Skills Programme checklist.

**Results:**

In the 34 eligible articles (*n* = 1,694 returned) two overarching themes emerged that dictated dietary habits in adolescents: Self-motivators and Uncontrollable factors. Gender differences arose whereby girls highlighted more external motivators (e.g., eat healthier, change dietary habits around boys and be thin to fit traditional norms) over their dietary habits. In contrast, boys focused on more internal motivators (e.g., gain autonomy, eat for enjoyment and pursue gains in physical performance). This suggests that motivation underlying how boys and girls eat differs. These trends were largely consistent across countries.

**Conclusion:**

Boys' and girls' food habits are not motivated by the same factors. To create more effective dietary interventions targeting health promotion, unique motivations behind food habits need to be understood and incorporated.

**Systematic review registration:**

Identifier: CRD42022298077.

## 1. Background

Dietary differences across sex (i.e., terms such as male and female rooted in biology) (1) and gender (i.e., terms such as man/boy or woman/girl as a part of self-identity) (2–4) groups are well-established in the literature (5–8). For example, consumption of health-protective foods such as produce (9–11) or meat alternatives (12, 13) is more common among females and women/girls, whereas males and men/boys report lower overall diet quality (9, 14–16). These differences in diet quality have been associated with an increased risk of chronic disease development in adult males compared to females (10, 17–19). However, greater instances of food restriction and poor emotional relationships with food (e.g., disordered eating) are more common among females and women/girls (19–21). This creates unequal risks for certain health trajectories based on sex and gender. To date, literature has mostly explored the role of sex in diet and health outcomes (22, 23). This is problematic as the influential role of social pressures that differ by gender is missing (8, 22, 23).

Adolescence (13–18 years) is a particularly advantageous age to understand the role of gender in relation to dietary habit formation. First, adolescents' dietary habits shift unfavorably compared to earlier childhood (14, 24–26), and gender differences emerge (14, 25, 27–31). Thus, adolescence marks a critical stage to understand why these unfavorable trends begin to emerge and has the potential for dietary interventions to have life course-altering impacts (10, 32). Second, self-identity, including gender identity, is a critical part of adolescence that is heavily influenced by social norms (33). This creates a situation where social norms and “fitting in” by eating a certain way may be at their peak. Finally, research has begun to explore the underlying factors that influence men's and women's dietary habits, but so far no research has consolidated these gender differences in adolescents (6, 34). At present, there is a gap in understanding how dietary habits can be optimally supported at a critical life stage (i.e., adolescence), where they can have lifelong impacts.

One aspect that may explain differences in boys' and girls' dietary habits is motivation (28, 35). In a cross-sectional study from the United States, girls expressed greater motivation to eat healthy (i.e., consuming produce and avoiding junk foods or sugary drinks) compared to boys (35). Other research delving deeper into *why* suggests that girls' motivation may be focused more on body shape (34, 36, 37) or fitting in by appearing healthy (25) more than boys. Theories such as self-determination theory (SDT) can be used to better understand these concepts, and in this case, SDT classifies these motivators among girls as more extrinsic, rooted in a desire to appease others (38). Inversely, intrinsic motivators rooted in personal enjoyment or beliefs in the importance of action (38) may better explain boys' dietary habits. In a review of the impact of sex, gender, and culture on dietary behaviors among adults, it was found that women may rely on extrinsic motivators (e.g., peers or emotions), whereas men rely more on intrinsic motivators like personal preference (8). These differences have yet to be confirmed in adolescents. Understanding these differences in motivation is important as different types of motivation can have different impacts on dietary behaviors (11, 35, 39–41).

Part of the inconsistencies in the data exploring gender, dietary habits, and motivation may be caused by the lack of context in underlying motivators inherent in quantitative studies. Furthermore, many studies exploring gender differences in motivation and eating have utilized higher income countries [e.g., the United States (28, 35, 36), Canada (25), Italy (11), and Portugal (40)]. As dietary habit (8, 23, 42, 43) and gender norms (4, 44, 45) can be influenced by culture and location, the data available thus far may not accurately represent the relationships between gender, motivation, and dietary habits in all places. Therefore, a systematic exploration of what motivates boys' and girls' dietary habits across diverse countries is needed to inform this understanding (25, 27, 28). As unfavorable shifts in adolescents' dietary habits can pre-dispose different groups to different negative health trajectories, identifying these motivators could have large impacts on health promotion (23). This is especially needed as no research has explored gender trends in adolescents' motivation behind eating (6, 34). Therefore, to help fill the gap in understanding how dietary habits can be optimally supported during adolescence, this study systematically reviewed differences in boys' and girls' perspectives on what motivates their dietary habits from diverse countries.

## 2. Methods

### 2.1. Study design

A systematic review was conducted in accordance with the Preferred Reporting Items for Systematic Reviews and Meta-Analyses (PRISMA) guidelines (46).

### 2.2. Participants and study criteria for inclusion

To be eligible, studies had to be available in English and explore adolescents' (13–18 years old) views on what impacted their dietary habits using qualitative methods. For the purpose of this review, dietary habits were defined as the quantity/quality of foods eaten or meal-related aspects. When age was not available, studies using 7th to 12th graders were eligible, as this range often coincides with adolescence. Studies that included younger (11–12 years) or older youth (19 years) were eligible if the study defined its population as “adolescents,” reported separate results, or had a substantial proportion of participants who were 13–18 years old. Adults could have also been participants as long as the results were presented separately. Studies had to utilize a mixed-gender sample (i.e., boys and girls) and report at least one unique theme by gender. In the absence of gender, biological sex was used. Studies conducted on adolescents living with a diagnosed psychological disorder (e.g., depression and eating disorders), pregnancy, severe dietary restriction (intake of two or more food groups), or allergies were not eligible. Dissertations, reviews, opinions, and editorials were excluded but hand-checked by AD.

### 2.3. Study protocol

This study was registered prior to search syntax formation with the International Prospective Register of Systematic Reviews (PROSPERO ID: CRD42022298077). All returned studies from the search in each database were uploaded into Covidence (47) where three trained research students screened the initial titles and abstracts. AD and one additional study team member had to select each study as eligible for retention at this stage and during full-text review. Once retained from the full-text review, data were extracted by a research team member and checked for accuracy by AD.

### 2.4. Search strategy

The search syntax for each database is found in Table 1. It was developed and pilot tested by the first author (AD) in consultation with a trained librarian.

**Table 1:** Search syntax in *n* = 4 databases.

**Database**	**Element**	**Search syntax**
Ovid MEDLINE	Group	Adolescent/or (adolescent^*^ or teen^*^ or youth or minor)
	Outcome	Food preferences/or (food^*^ or eating) adj (choice^*^ or choose^*^ or preference^*^ or meaning^*^) or eating/or feeding behavior/
	Contrast	Man/or women/(girl^*^ or boy^*^ or female^*^ or male^*^ or gender or sex)
	Methods	Qualitative research/or (qualitative^*^ or interview^*^ or diar^*^ or journal^*^ or ethnography^*^)
Web of Science	Group	adolescent^*^ or teen^*^ or youth or minor
	Outcome	(Food^*^ or eating or diet^*^) near/2 (choice^*^ or choose^*^ or preference^*^ or meaning or view^*^ or perspective^*^ or norm^*^)
	Contrast	Boy^*^ or girl^*^ or female^*^ or male^*^ or gender^*^ or sex^*^
	Methods	Qualitative^*^ or interview^*^ or “focus group^*^”
CINHAL	Group	(MH “Adolescence+”) or adolescent^*^ or teen^*^ or minor^*^ or youth^*^
	Outcome	MH “food preferences”) or (food^*^ or eating or diet^*^) N2 (choice^*^ or choose^*^ or preference^*^ or meaning or view^*^ or perspective^*^ or norm^*^)
	Contrast	(MH “male”) or (MH “female”) (boy^*^ or girl^*^ or female^*^ or male^*^ or gender^*^ or sex^*^)
	Methods	(MH “qualitative studies+”) or (qualitative^*^ or interview^*^ or focus group^*^)
Embase	Group	Adolescent/or (adolescent^*^ or teen^*^ or youth or minor^*^)
	Outcome	Food preference/or (food^*^ or eating or diet^*^) adj2 (choice^*^ or choose^*^ or preference^*^ or meaning^*^ or view^*^ or motivate^*^ or perspective^*^ or norm^*^)
	Contrast	Boy/or girl/or (boy^*^ or girl^*^ or female^*^ or male^*^ or gender^*^ or sex^*^)
	Methods	Qualitative research/or (qualitative^*^ or interview^*^ or focus group^*^)

MH or word: Recognized medical subject heading (MeSH). Adj#, N# or near/#: Within the specified number (#). ^*^: Truncated word ending. CINHAL: Cumulated Index to Nursing and Allied Health Literature.

### 2.5. Data sources

MEDLINE Ovid, Embase, Web of Science, and the Cumulative Index to Nursing and Allied Health Literature (CINHAL) were searched from conception to 17 December 2021. To be considered, studies had to discuss adolescents' motivations behind their food habits.

### 2.6. Data extraction

The three research assistants extracted study data (e.g., title, country, aims, data collection methodology, sample characteristics, and main themes). The fidelity of all extracted data was checked by AD.

### 2.7. Data analysis

A thematic analysis was led by AD in conjunction with CB (48). The two iteratively derived the codebook by reviewing all extracted themes and inductively grouping themes together (49). Discrepancies were resolved with TC. Themes could have been stated uniquely among boys in some studies, girls in others, or as non-gender unique (i.e., said by boys and girls in a study). To interpret this, this review analyzed differences in the themes uniquely reported by boys or girls in any of the 34 studies. A sub-analysis of themes across countries was also conducted by AD, and trends were considered by country.

The quality and potential for bias of all included studies were assessed using the Critical Appraisal Skills Programme (CASP) checklist (50, 51). This appraisal tool consists of 10 questions. In the appraisal, one research assistant and CB independently reviewed studies and chose “yes,” “no,” or “somewhat” to each question. Discrepancies between the two were resolved by AD. As the CASP program does not have a formal scoring system, this study created one (yes = 2, somewhat = 1, and no = 0). Calculated scores were divided by the maximum score of 20 to obtain a percent out of 100. Values > 70.0% were considered high quality, 40.0–60.0% as moderate, and <40.0% as low (52).

## 3. Results

**Figure 1:**
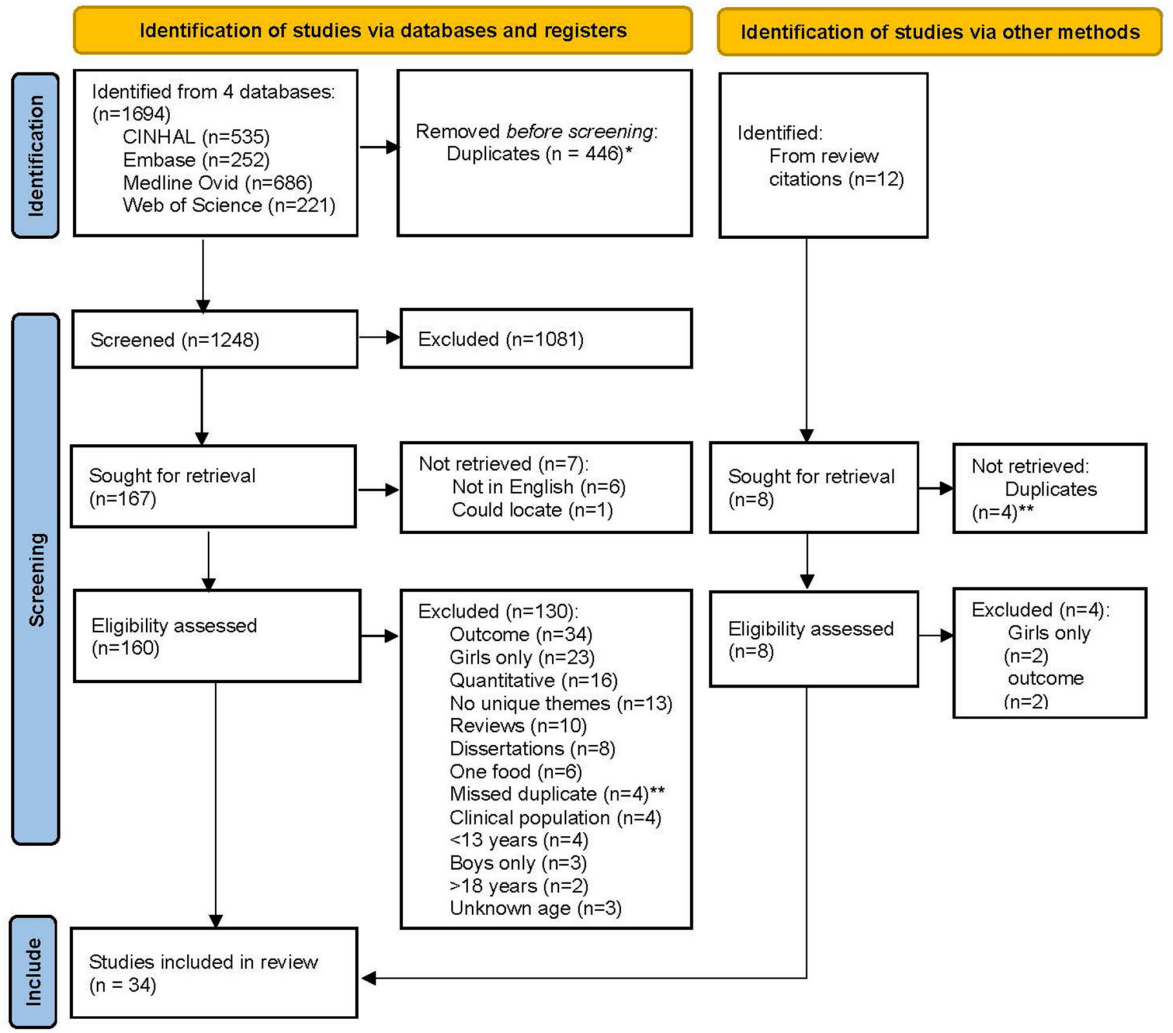
PRISMA flow diagram of all included and excluded studies with justification. *Duplicates automatically removed by the Covidence software program. **Duplicates manually removed by the research team.

A total of 1,694 studies were originally returned. Of these, 446 were duplicates and were removed by Covidence. This left 1,248 records that were screened, with 160 moving to full-text review. Thirty of these were found to be eligible. After searching the reference lists of returned reviews, 12 studies were identified, and four met eligibility (*n* = 34). See Figure 1 for a flow diagram. All extracted data from each study are shown in the Supplementary material.

### 3.1. Participant characteristics

Six studies (17.6%) were conducted in the United States (53–58). Three were conducted in China (8.8%) (59–61). Australia (62, 63), Costa Rica (64, 65), Bangladesh (66, 67), Ireland (68, 69), and Sweden (70, 71) all had two studies (5.9%) each. Ethiopia (72), India (73), Indonesia (74), Iran (75), Morocco (76), Netherlands (77), Peru (78), Scotland (79), Slovakia (80), South Africa (81), United Arab Emirates (82), and United Kingdom (83) all had one study (2.9%) each. One study considered data from Canada and India, these countries were evaluated separately (84). Sub-analyses were grouped by continent and income level classified by the World Bank (85) as there were too few countries to draw country-level trends.

Sample sizes ranged from 12 to 141 participants, with the majority disclosing how many boys and girls participated (79.9%). Of the studies disclosing how many boys and girls participated, 55.2% included more girls than boys (55–59, 62, 68, 70–74, 77, 83, 84, 86) and 24.0% included an equal number of boys and girls participated (54, 60, 63, 67, 76, 79, 82). One study did not indicate specific participant numbers or genders (75).

Four studies reported only participants grades (11.8%) (53, 56, 60, 82). All other studies reported age ranges from 8 to 19 years (25, 54, 55, 58, 59, 61–66, 68–73, 76–81, 83, 84, 86). Of these, 14 studies (46.7%) included ages below 13 years (25, 58, 59, 62–64, 68–70, 72, 80, 81, 83, 84), one study included adolescents over 18 years, and 4 studies (13.0%) included older and younger ages (57, 67, 74, 75).

### 3.2. Study methods

The majority of studies (67.7%) used focus groups (53, 55–60, 62, 64–66, 68–73, 75–77, 81, 83, 84), with 30.4% of these conducting mixed-gender groups (55, 56, 58, 60, 70, 71, 77). Parents were included in 26.1% of studies (53, 65, 66, 72, 73, 76, 81), but only present with adolescents during data collection in one study (53). Semi-structured interviews (26.5%) (25, 54, 61, 63, 67, 78, 79, 82, 86), open ended text (2.9%) (80), and immersed field observation (2.9%) (74) were also used. Less than half of the studies reported the theoretical lens used to interpret the collected data (41.2%) with a Socio-Ecological Model used most (20.6%) (25, 53, 58, 61, 64, 76, 78).

### 3.3. Quality appraisal

Quality appraisal scores ranged from 45 to 85%, with a mean score of 66%. Thirteen studies (38%) ranked as high quality (scores ≥ 70%) (56, 58, 59, 62, 66, 70–72, 74, 78–81), while all other studies ranked as moderate. Most studies did not report details on researcher relationships.

### 3.4. Adolescents' rationale behind their dietary habits

Two overarching themes dictated adolescents' dietary habits, including what, when, or how much to eat, regardless of gender. These two themes included Self-motivators and Uncontrollable factors. *Self-motivators* spoke to adolescents identified intrinsic and extrinsic motivators that they perceived some level of control over. Six sub-themes (*Manage health, Enjoyment, Fit in with peers, Manage performance, Control appearance*, and *Form a food identity*) arose. Adolescents also recognized *Uncontrollable factors*, factors they perceived they had no control over that limited their dietary habits. This theme included two sub-themes: *Cognitive control and Logistics*. A summary and explanation of these themes are found in Table 2.

**Table 2:** Emergent themes and their sub-themes in *n* = 34 studies.

**Theme**	**Sub-theme**	**Explanation**
Self-motivators	Manage health	Altering of dietary habits for perceived health benefits. This included avoiding conditions of poorer health such as feeling sick, chronic diseases, or excessive weight gain. The importance of food quantity and quality for health was also exclusively highlighted.
	Enjoyment	Sensory factors such as taste, smell, texture, or visual appeal of foods or innate preferences regardless of sensory properties (e.g., preference for junk foods) motivated dietary habits.
	Fit in with peers	Desire to be socially accepted by peers motivated dietary habits. This resulted in altered food habits in the presence of peers to fit in and be accepted.
	Manage performance	Physical aspects of performance for athletics or improving energy and mental performance were discussed as motivators for eating.
	Control appearance	Growing bigger, being thin, or managing aesthetics (e.g., preventing acne) regardless of health implications motivated dietary habits.
	Form a food identity	Adolescents discussed the importance of forming a consistent food identity, while still acknowledging the role of parents.
Uncontrollable factors	Logistics	Aspects of the physical environment in homes, schools, or communities (e.g., cost, availability, rules), family schedules, or limited food skills (e.g., knowing how to cook) dictated dietary habits beyond adolescents' perceived control.
	Cognitive eating	Factors adolescents perceived to have no control over that originated from within them such as emotional eating or uncontrollable cravings impacted dietary habits.

**Figure 2:**
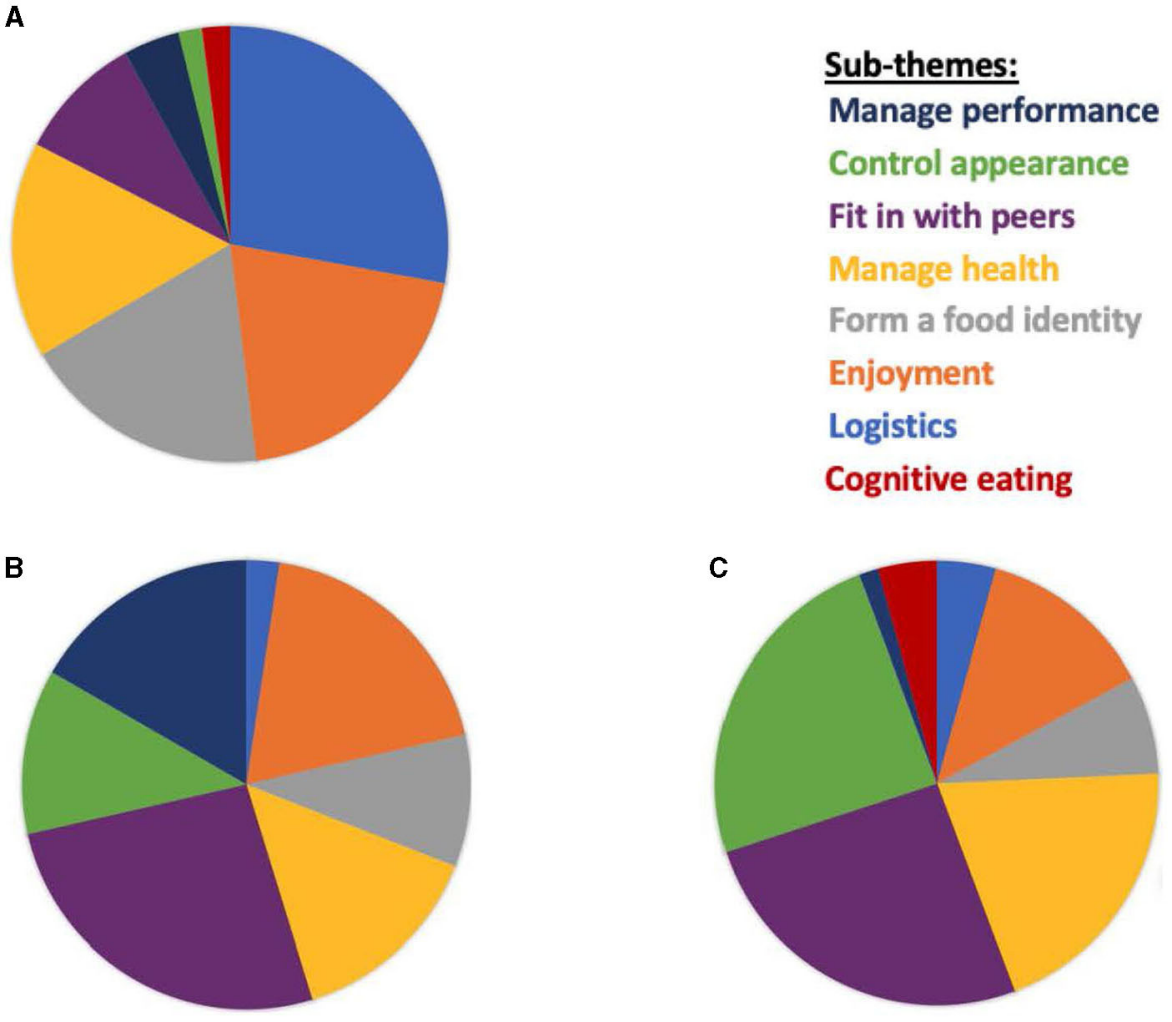
Differences in motivation behind boys ‘and girls' dietary habits. **(A)** Stated by boys and girls (non-gender unique). **(B)** Gender-unique themes stated by boys. **(C)** Gender-unique themes stated by girls.

In studies reporting non–gender-unique influences (i.e., said by boys and girls in the same study), *Logistics* was found to be the largest factor impacting dietary habits, accounting for 28.0% of the stated themes in the 34 studies. This was followed by *Enjoyment* (20.0%), *Form a food identity* (19.0%), *Manage health* (16.0%), *Fit in with peers* (9.0%), *Manage performance* (4.0%), *Control appearance* (2.0%), and *Cognitive control* (2.0%). This hierarchy was not the same among boys and girls when it came to unique gender themes stated in individual studies (see Figure 2).

Boys and girls both uniquely identified *Fit in with peers* the most (26.0% of themes in each group). Among boys, this was closely followed by *Enjoyment* (19.0%), *Mange performance* (17.0%), *Manage health* (14.0%), *Control appearance* (12.0%), *Form a food identity* (10.0%), and *Logistics* (2.0%). *Cognitive control* was not uniquely stated by boys in any of the 34 studies. This differed from girls, where *Control appearance* was the second most stated motivator (24.0%) followed by *Manage health* (20.0%), *Enjoyment* (13.0%), *Form a food identity* (7.0%), *Cognitive control* (4.0%), *Logistics* (4.0%), and *Manage performance* (2.0%).

Unique gender differences in stated sub-themes are discussed below, with an exploration by country. A summary of unique gender sub-themes by country is found in Table 3.

**Table 3:** Unique gender emergent themes by country (*n* = 34 studies).

	**Form a food identity**	**Control appearance**	**Manage health**	**Manage performance**	**Fit in with peers**	**Enjoyment**	**Cognitive eating**	**Logistics**
**Africa**								
Ethiopia (*n* = 1)						B, G		
Morocco (*n* = 1)		G	G					B
South Africa (*n* = 1)			G					G
**Asia**								
Bangladesh (*n* = 2)	B, G	B	G		B	B, G		
China (*n* = 3)		B, G				B		
India (*n* = 2)^*^			B					
Indonesia (*n* = 1)					G			
Iran (*n* = 1)		G	B, G	B, G	G	G		
United Arab Emirates (*n* = 1)					B, G			
**Europe**								
Ireland (*n* = 2)	G	G	B, G		G	B	G	
Netherlands (*n* = 1)			G	B				
United Kingdom (*n* = 1)			G	B	G			
Scotland (*n* = 1)					B, G	B, G		
Slovakia (*n* = 1)	B	B, G		B	B			
Sweden (*n* = 2)			B		G		G	G
**North America**								
Canada (*n* = 3)^*^	B, G	B, G	B, G	B	B, G			
United States (*n* = 6)	G	B, G	G		B, G	G	G	G
**Oceania**								
Australia (*n* = 2)			B			B		
**South America**								
Costa Rica (*n* = 2)	G	G		B	B, G			
Peru (*n* = 1)		G						

B: Unique emergent theme among boys. G: Unique emergent theme among girls. n: Number of studies. ^*^From the same study comparing two countries.

#### 3.4.1. Self-motivators: an exploration by gender and country

##### 3.4.1.1. Manage health

Boys emphasized *Managing health* through diet quantity in four studies, whereas girls did not mention Managing health through diet quantity in any of the 34 studies. Instead, girls uniquely identified diet quality (*n* = 2 studies) (65, 75), avoiding disease (*n* = 2 studies) (75, 87), feeling sick (*n* = 1 study) (76), and avoiding excessive weight gain (*n* = 7 studies) (65, 66, 75, 77, 83, 84, 87). Both groups highlighted how pairing unhealthy foods with healthy foods canceled out negative impacts [*n* = 1 study boys (86); *n* = 2 studies girls (68, 81)]. These trends presented no obvious dependence on the country.

##### 3.4.1.2. Enjoyment

Meat (*n* = 1 study) (60) and unhealthy foods (*n* = 1 study) (69) were expressed as key foods sought out by boys. In contrast, girls uniquely stated food smell (*n* = 1 study) (58) as a motivating factor for dietary habits. Both groups uniquely highlighted preferences for food outlets [*n* = 1 study boys (73); *n* = 1 study girls (79)] and food visuals [*n* = 3 study boys (59, 63, 72); *n* = 2 studies girls (58, 75)]. Within this, boys specifically talked about the motivating visual role of food advertisements (*n* = 2 studies) (59, 63), whereas girls only discussed visual aspects of foods in-person. No apparent trends by country arose though both studies uniquely identified food advertisements among boys from high-income countries (China and Australia).

##### 3.4.1.3. Fit in with peers

*Fitting in with peers* was the most often stated dietary motivator, uniquely among boys and girls. However, Fitting in with peers was acknowledged differently by boys and girls. First, when discussing school lunches, boys spoke about peer pressure to be active at lunch instead of eating (*n* = 2 studies) (25, 79). In contrast, girls highlighted peer pressure to chat instead of eating lunch (*n* = 2 studies) (25, 79). Girls also identified eating healthier in the presence of their peers to gain social acceptance in five studies (64, 65, 68, 74, 84), altering food habits in the presence of boys to appear more attractive (*n* = 5 studies) (53, 65, 67, 69, 83), and avoiding weight stigma by eating certain foods (*n* = 1 study) (75). Boys did not identify altering their dietary habits as a motivator to fit in in these ways. Both groups uniquely identified eating worse to fit in with peers, though boys discussed this more often [*n* = 1 (82) study vs. *n* = 3 (64, 65, 79) studies, respectively]. Finally, both groups discussed the importance of appearing masculine [boys *n* = 4 studies (58, 64–66)] or feminine (girls *n* = 3 studies) (64, 65, 75) by altering food quantity or quality around peers.

All continents had countries that suggested *Fitting in with peers* as a gender-unique motivator except for studies from Africa. Furthermore, attracting a partner by using food among girls was more prevalent in higher income countries (the United States, Ireland, and the United Kingdom) compared to lower-income countries (stated in Costa Rica). Attempts to adhere to gender norms in dietary habits, including appearing masculine or feminine, were recognized in lower-income countries (Bangladesh, Iran, and Costa Rica) by boys and girls, but only among boys in a high-income country (the United States).

##### 3.4.1.4. Manage performance

In five studies, boys uniquely identified a desire to increase athletic performance as a motivating factor (25, 64, 75, 77, 83). Girls did not uniquely identify this factor in any of the 34 studies, but both groups did suggest the importance of energy and mental performance in eating decisions [*n* = 2 studies boys (25, 80); *n* = 1 study girls (75)]. Regarding increasing athletics, boys stated this motivator in higher income countries (the Netherlands, United Kingdom, and Canada) and lower income countries (Costa Rica and Iran). No emphasis on *Manage performance* as a unique gender motivator (athletics, energy, or mental) was stated in studies from Africa.

##### 3.4.1.5. Control appearance

Altering appearance irrespective of health outcomes was commonly discussed in the 34 studies as a key motivator among all adolescent groups. However, boys and girls talked about this very differently. Boys discussed a desire to grow in size or stature (*n* = 5 studies) (25, 57, 59, 66, 80), whereas girls expressed desires to actively try and be thin by using food (*n* = 14 studies) (25, 53, 55, 59, 61, 64, 65, 68, 69, 75, 76, 78, 80, 86). Aesthetic aspects related to hair or the skin were also identified uniquely by girls in three studies (64, 68, 75). In African (Morocco) and South American (Costa Rica and Peru) countries, body shape as a motivator was only uniquely identified by girls. On all other continents, boys and girls mentioned body shape as a motivator.

##### 3.4.1.6. Form a food identity

Adolescent boys and girls differed in how they discussed balancing parental influence on their dietary habits. Boys were motivated to gain control from parents (*n* = 1 study) (25) by making autonomous food decisions, whereas girls suggested continuing to adopt some family practices (*n* = 1 study) (65). Boys also highlighted wanting to be unique (*n* = 1 study) (25) and form habits (*n* = 1 study) (80) that were their own, whereas girls were motivated to seek out opportunities that facilitated gains in food-related skills, such as nutrition or cooking knowledge (*n* = 1 study) (58). Both groups discussed how their dietary habits were motivated by factors they self-valued, such as animal welfare (*n* = 1 study) (69) and religion (*n* = 1 study) (67) among girls and culture (*n* = 1 study each) among both (67). Forming a food identity did not arise as a unique gender self-motivator in countries from Africa (Ethiopia, Morocco, or South Africa).

#### 3.4.2. Uncontrollable factors: an exploration by gender and country

##### 3.4.2.1. Cognitive eating

Emotional eating arose as a unique stated influence on eating among girls in three studies (54, 68, 70) from North America (United States) and Europe (Ireland and Sweden). Boys did not uniquely refer to this factor in any of the 34 studies. No studies from Africa (Ethiopia, Morocco, and South Asia), Oceania (Australia), or South America (Costa Rica and Peru) indicated that boys or girls uniquely identified cognitive eating as a dietary influence.

##### 3.4.2.2. Logistics

Both boys and girls uniquely talked about the role of time and busy schedules in dictating their dietary habits (*n* = 1 study for each) (70, 76). Girls further spoke to how cost (*n* = 1 study) (58) and a perceived lack of food-related skills due to their age or available experiences (*n* = 1 study) (81) confined dietary habits. Logistics arose as unique gender themes among boys and girls in Africa (Morocco and South Africa) and by girls in Europe (Sweden) and North America (United States).

## 4. Discussion

**Figure 3:**
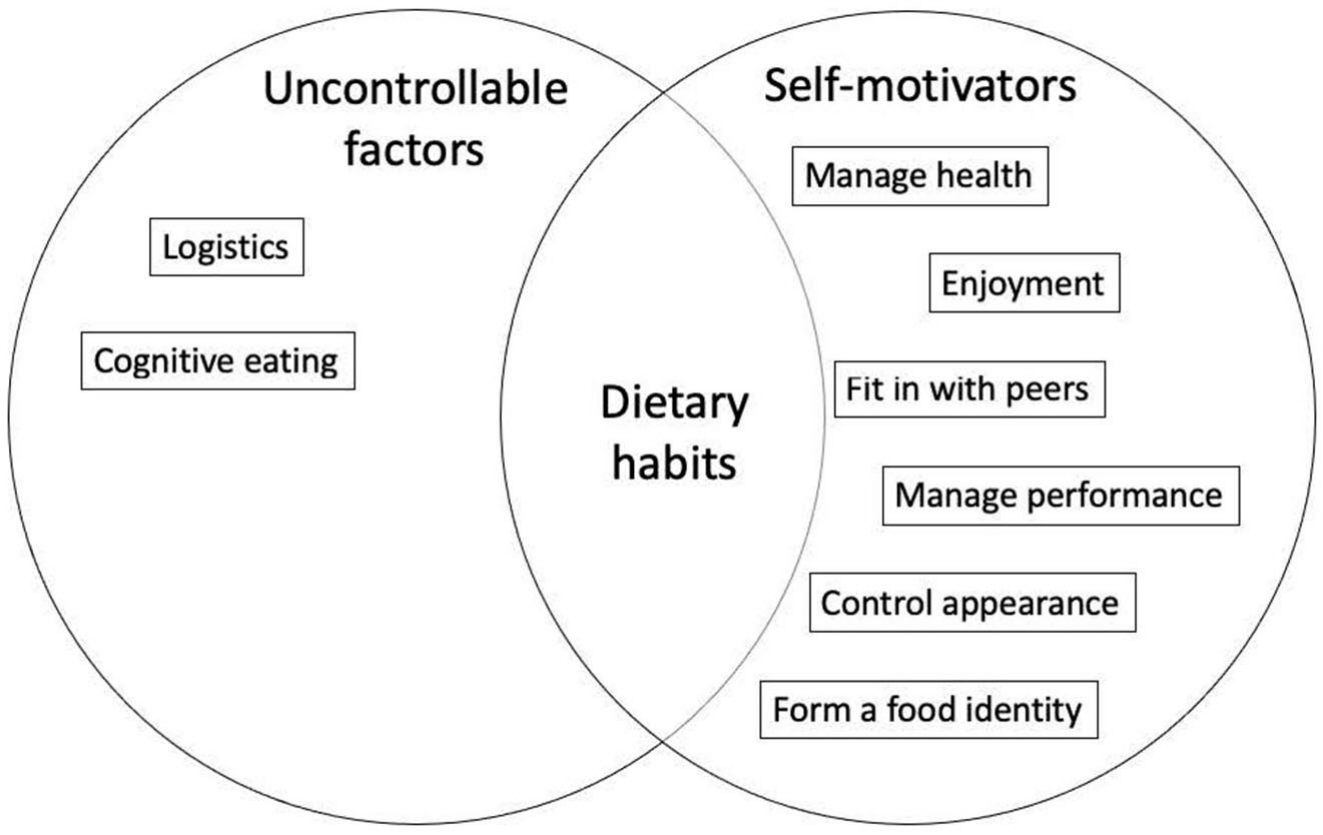
Summary of emergent themes in *n* = 34 qualitative studies that boys and girls felt motivated their dietary habits. n: Number of studies.

This systematic review consolidated trends in boys' and girls' motivations behind their dietary habits. Findings suggest that adolescents are *Self-motivated* by many diverse factors and also feel that there are further *Uncontrollable factors* that confine their dietary habits (Figure 3). Gender differences arose in all self-motivators and in uncontrollable factors that had a cognitive tie (i.e., emotional eating). Trends were largely persistent, regardless of country or continent.

### 4.1. Self-motivators, gender, and dietary habits

Though boys and girls both highlighted the importance of *Fitting in with peers*, they did so in different ways, a finding that is similar to other reviews (16, 88). In this study, we found that girls discussed how peers contributed to eating healthier and how they altered their dietary habits in front of boys to appear attractive. The boys did not mention these influences. It is possible that girls are more aware of gender connotations surrounding certain foods (e.g., healthy food as being more feminine), and thus consume them to alter social perceptions more so than boys (89). Yet, only three studies in this review saw girls explicitly acknowledge gender norms surrounding food, and furthermore, boys in this review also identified this (*n* = 4 studies). This may suggest instead that adolescent girls' dietary motivation is less about appearing feminine and more about fitting in socially compared to boys, which matches adult literature where women express greater importance on aligning with social norms compared to men (7). However, girls' emphasis on *Concern for health* (*n* = 11 studies) or *Food quality* in the context of health matches historical gender norms in other literature (4, 34, 90, 91). Taken together with the motivation to fit in by eating healthier with peers, it seems girls may actually still be attempting to conform to a version of femininity, it is just not acknowledged overtly (3). Gender literature has called for more public awareness of how gender norms still influence many behavioral facets, such as careers and parenting roles (3, 4), and it appears dietary habits are no exception. Future research needs to untangle the extent to which gender norms covertly impact boys' and girls' motivation behind their dietary habits (e.g., are girls more concerned about health or do they just say this because they feel they “should”?) to address how gender norms may be controlling eating (3, 4).

Overall, boys perceived self-motivators that were more intrinsically focused than girls, a finding that aligns with adult literature (8). For example, boys' desire to grow, manage physical performance, eat foods they personally enjoy, and form a food identity that is autonomous suggests more intrinsic motivation. These contrast girls wanting to be thin, managing aesthetics, and altering dietary habits in front of peers/boys to be accepted. Though the trends in body shape found in this review are not original (27, 29, 92, 93), our study contributes to understanding the larger picture of adolescents' motivation behind eating based on gender. Specifically, we call on researchers who create dietary interventions targeting adolescents to consider focusing on using language and content that speaks to gender-diverse motivators, including body shape, fitting in, and pursuing autonomy (e.g., focus on autonomy and personal control among boys vs. highlighting social relationships and pressures among girls) (6, 7, 10, 23, 88). We also call on research to clarify the role of athletic performance in adolescents' dietary choices, as this review only identified this influence among boys. Other literature has suggested that dietary impacts among boys and girls vary based on the level of competition (21, 94, 95), whereas literature among adults has suggested that men may be more encouraged to alter their diet in regard to physical activity in less competitive contexts (8). Confirming the relationship between physical activity, competition level, and diet will help inform if interventions seeking to alter these health behaviors should combine them or keep them separate, and for whom.

### 4.2. Uncontrollable factors, gender, and dietary habits

Boys and girls did not differ in their unique perception of the role of logistics outside of their control. This is somewhat surprising given that literature suggests boys are more often granted more behavioral autonomy compared to girls (3, 43, 96). The findings of this review may thus indicate that parent's food-related activities (e.g., rules, what is brought into the home) may not be based on their child's gender as much as previously thought.

Our findings that only girls identified *Cognitive eating* (e.g., emotional eating) as an uncontrollable motivator may suggest that biological differences play a significant role. Research examining appetite (97, 98) and coping strategies for stress (99, 100) has found sex differences, largely through hormones. These differences in hormones can impact eating through the gut–brain axis, having a role in intentional eating for cognitive aspects compared to hunger (101). A recent cross-sectional study exploring mothers' and fathers' parenting practices on boys' and girls' dietary habits further supports some role for differences in cognitive factors, as it found that adolescents' dietary behaviors were dependent on their gender only through differences in their motivation and self-efficacy (28). Additional research in adults has found that attitudes toward emotional eating vary between women and men, with women expressing more enactment (5). Future sex and gender-based analyses are needed to establish if gender and social norms, cognitive or hormonal factors rooted in biology (or both), ultimately guide dietary habits. This knowledge could inform future dietary interventions focused on health promotion through diet (10, 23).

### 4.3. Trends across countries

Major trends across countries themselves did not arise. This is likely because a limited number of countries were present in this review, and even less had multiple studies conducted. This matches data from a single study exploring the underlying motivation behind adults' dietary habits, which found prevailing trends across the 23 included countries between men and women (34). However, across continents, some small trends did emerge and may reflect trends based on a country's overall income level.

Studies from Africa and East Asia in this review did not uniquely identify any influences related to *Forming a food identity, Managing performance*, or *Fitting in with peers*. This may suggest that boys and girls do not experience pressures to be different (e.g., autonomy among boys) or match a group (e.g., match peers) based on typical gender trends seen in other countries. It is not clear why, but it could be attributed to differences in cultural norms across regions. Emotional eating also arose only unique among girls in high-income countries in Europe and North America. This could suggest that eating based on emotions is tied to aspects of socio-economic status, such as greater food availability or marketing. More studies and an exploration of dietary habits across diverse countries are needed to solidify our understanding of these concepts.

### 4.4. Limitations

Many studies in this review focused on eating intentions, and this could have created a gap in understanding adolescents' practiced habits. Furthermore, most studies were from high-income white nations. The findings could overrepresent the lens of these groups. They may also underrepresent the influences that impact boys' dietary habits, as majority of studies recruited more girls than boys. Most studies (76.7%) relied on focus groups, which are subject to responder bias. Finally, literature thus far often has relied on dichotomized sex (e.g., male/female), leaving out the experiences of gender-diverse folks (102). Due to this, we are unable to explore any trends in motivation and dietary habits among gender-diverse adolescents.

## 5. Conclusion

Adolescents' dietary habits are ruled by *Self-motivators* and *Uncontrollable factors* outside their perceived control. Girls specifically emphasized more external motivators (e.g., eat healthier, change dietary habits around boys, and be thin to fit traditional norms) compared to internal motivators in boys (e.g., gain autonomy, eat for enjoyment, and pursue gains in physical performance). This suggests that the motivation underlying *how* boys and girls eat differs and is largely consistent regardless of country. Health promotion endeavors targeting diet should thus incorporate gender-unique motivators behind eating to support their likelihood of success (6, 7, 19). Future studies are also needed to fully untangle how both gender and sex impact adolescents' dietary habits (10, 23).

## Data availability statement

The original contributions presented in the study are included in the article/Supplementary material, further inquiries can be directed to the corresponding author.

## Author contributions

AD: Conceptualization, Data curation, Formal analysis, Investigation, Methodology, Project administration, Resources, Software, Supervision, Validation, Visualization, Writing—original draft, Writing—review and editing. CB: Formal analysis, Investigation, Writing—review and editing. TC: Conceptualization, Resources, Supervision, Validation, Writing—review and editing.
