# Supplementary material for: Boys and girls differ in their rationale behind eating: a systematic review of intrinsic and extrinsic motivations in dietary habits across countries
Source: Front Nutr. 2023 Sep 29;10:1256189. doi: 10.3389/fnut.2023.1256189 (PMC10570531; doi:10.3389/fnut.2023.1256189)
Supplement: Supplementary file 1 [file Table_1.DOCX]

| Author/Year | Country | Research Aims | Data collection | n (% girls) | Age* | Theoretical lens | Quality score** |
| --- | --- | --- | --- | --- | --- | --- | --- |
| **Africa (n=3)** | | | | | | | |
| Abera  2020 | Ethiopia | Explore influences on adolescent diet and physical activity. | Focus groups | 41  (58.5%) | 10-17^a^ |  | 0.7 |
| El-Ammari 2020 | Morocco | Identify the prevalence of unhealthy dietary behaviours and their social-ecological influences in adolescents. | Focus groups | 56  (50.0%) | 14-16^a^ | Socio- Ecological Model | 0.60 |
| Wrottesley 2019 | South Africa | Understand facilitators and barriers to healthy eating practices and physical activity in younger and older urban adolescent boys and girls in South Africa. | Focus groups | 38  (47.4%) | 10-17^a^ |  | 0.70 |
| **Asia (n=9)** | | | | | | | |
| Islam  2019 | Bangladesh | Explore adolescents’ and mothers’ perception of broader sociocultural aspects that sculpt the food choices, eating habits and physical activity behaviors of adolescents in Matlab, Bangladesh. | Focus groups | 28 | 14-17^a^ | Social Constructivist | 0.70 |
| Khan  2014 | Bangladesh | Explore how the adolescents identify diet and why do they select these types of foods as diet and how they conceptualize health. | 1-on-1 interviews | 32  (50.0%) | 10-19^a^ |  | 0.55 |
| Chan  2016 | China | Explore snacking behavior and perspectives on healthy and unhealthy food choices among adolescents in Mainland China. | Focus groups | 24  (58.3%) | 12-13^a^ |  | 0.75 |
| Siu  2019 | China | Investigate the barriers to the adoption of healthy eating habits among secondary school students from low-income families in Hong Kong. | Focus groups | 30  (50.0%) | 7-12^b^ |  | 0.65 |
| Veeck  2014 | China | Examine the major influences of food choices of Chinese teenagers within a dynamic food marketing environment | 1-on-1 interviews | 16  (37.5%) | 15-19^a^ | Socio- Ecological Model | 0.45 |
| Joshi-Reddy, 2020 | India | To explore the perceptions of adolescents and their caregivers on drivers of diet and physical activity in rural India in the context of ongoing economic, social and nutrition transition. | Focus groups | 38 (52.6%) | 15-17^a^ | Gender | 0.65 |
| Roshita 2021 | Indonesia | Describe the dietary and physical activity behaviors of Indonesian adolescent girls and boys and to understand related influencing factors. | Immersed field observation | 26  (57.7%) | 12-19^a^ |  | 0.80 |
| Peykari 2011 | Iran | Study the adolescence opinions among nutritional habits and beliefs. | Focus groups |  | 10-19^a^ |  | 0.60 |
| Makansi 2018 | United Arab Emirates | Describe eating behaviours of adolescents in Dubai and the factors associated with fruit and vegetable intake. | 1-on-1 interviews | 14  (50.0%) | 11-12^b^ |  | 0.60 |
| **Europe (n=8)** | | | | | | | |
| Crofton 2014 | Ireland | Examine Irish teenagers’ perceptions towards healthy  and unhealthy snack foods and to identify the factors influencing healthy and unhealthy snacking behaviours. | Focus groups | 113 (54.9%) | 11-18^a^ |  | 0.65 |
| Stevenson 2007 | Ireland | Examine potential conceptual, physical, individual, developmental and social barriers to healthy eating in focus group discussions with adolescents. | Focus groups | 75  (46.7%) | 12-15^a^ |  | 0.60 |
| Hermans 2017 | Netherlands | Understand adolescents’ food and health perceptions and their willingness to be involved in a specific school-based prevention program. | Focus groups | 42  (59.5%) | 13-16^a^ |  | 0.65 |
| Calvert 2020 | United Kingdom | ﻿Explore the perceived psychosocial influences associated with healthy and unhealthy food choices amongst secondary school students living in socially deprived areas in England. | Focus groups | 46  (54.4%) | 11-13^a^ |  | 0.65 |
| Wills  2005 | Scotland | Explore the secondary school environment as a context for understanding young teenagers' food and eating practices. | 1-on-1 interviews | 36  (50.0%) | 13-14^a^ |  | 0.70 |
| Stefanova 2018 | Slovakia | Provide insight into children's views regarding the most critical areas of health behaviour using qualitative analysis. | Open ended worksheets | 26  (46.2%) | 12-15^a^ |  | 0.75 |
| Jonsson 2017 | Sweden | Illuminate factors that undermine the healthy habits of adolescents from a multicultural community with low socioeconomic status in Sweden with regard to physical activity and food, as stated in their own voices. | Focus groups | 53  (60.4%) | 12-13^a^ | Gender | 0.85 |
| Rendahl 2018 | Sweden | Explore and elucidate adolescents' reasoning about risks related to food and eating. | Focus groups | 31  (64.5%) | 15-16^a^ | Risk profiling | 0.75 |
| **North America (n=8)** | | | | | | | |
| Deslippe 2021 | Canada | Understand gender differences in boys' and girls' dietary behaviors during the transition to secondary school. | 1-on-1 interviews | 27  (48.2%) | 12-14^a^ | Socio- Ecological Model | 0.60 |
| McPhail 2011 | Canada | Examine how class influenced teen participants’ moral judgments about fast food environments and consumption, and how their moral boundary marking concerning fast food related (or not) to class location or identity. | 1-on-1 interviews | 132 (58.3%) | 13-19^a^ |  | 0.6 |
| Bauer  2004 | United States | Identify factors in middle school social and physical environments that support or interfere with efforts to promote healthful nutrition and physical activity. | Focus groups | 26 | 7-8^b^ | Socio- Ecological Model | 0.65 |
| Beck  2019 | United States | Explore barriers and facilitators to healthy eating among low-income Latino adolescents. | 1-on-1 interviews | 30  (50.0%) | 13-17^a^ | Capability, opportunity, motivation, behaviour theory | 0.65 |
| Campbell 2009 | United States | Determine the independent food choices of urban minority adolescents and Hispanic teens in particular, and their knowledge of nutrition and health related to these food selections. | Focus groups | 12  (91.7%) | 14-16^a^ |  | 0.60 |
| Kubik  2005 | United States | Understand factors that may influence the dietary and physical activity practices of adolescents attending an alternative high school. | Focus groups | 70  (51.4%) | 9-12^b^ |  | 0.70 |
| Neumark-Sztainer 1999 | United States | Assess adolescents' perceptions about factors influencing their food choices and eating behaviors. | Focus groups | 141 (68.1%) | 12-19^a^ |  | 0.65 |
| Swanson 2013 | United States | Elicit perspectives on healthful eating from youth residing in [central Appalachia] under-resourced region. | Focus groups | 68  (54.4%) | 8-17^a^ | Socio- Ecological Model | 0.75 |
| **Oceania (n=2)** | | | | | | | |
| Ronto  2016 | Australia | Explore adolescents' perspectives of the importance of food literacy on their dietary behaviours. | Focus groups | 131 (68.7%) | 12-17^a^ |  | 0.80 |
| Thaichon 2016 | Australia | Investigate the impact of online advertising on social network sites on children’s intention to consume fast food in Australia | 1-on-1 interviews | 30  (50.0%) | 11-16^a^ |  | 0.55 |
| **South America (n=3)** | | | | | | | |
| Monge-Rojas  2005 | Costa Rica | Assess the perceptions of rural and urban Costa Rican adolescents about their diet and the factors they consider significant to healthful eating. | Focus groups | 108 | 12-18^a^ | Socio- Ecological Model | 0.65 |
| Monge-Rojas  2015 | Costa Rica | Identify the influence of gender stereotypes on eating habits among Costa Rican adolescents. | Focus groups | 92 | 14-17^a^ | Social cognitive theory | 0.60 |
| Banna 2016 | Peru | Gain insight into socio-cultural influences on eating in adolescence. | 1-on-1 interviews | 14  (42.9%) | 15-17^a^ | Socio- Ecological Model | 0.75 |
| **Across Continents (n=1)** | | | | | | | |
| Correa 2017 | India/ Canada | Identify perceptions and attitudes towards healthy eating amongst adolescents of Indian origin in two countries. | Focus groups | 73  (53.4%) | 11-18^a^ | Health belief model | 0.65 |

n=number. %=percent.

*Age presented as years denoted by ^a^ or grade level denoted by ^b^.

^**^Quality appraisal scores calculated using the Critical Appraisal Skills Program (CASP) Checklist. Maximum score of 20.
